# Acupuncture in Uterine Fibroids Management: Literature Update on Frequently Used Acupoints and the Underlying Mechanism—A Narrative Review

**DOI:** 10.1002/hsr2.71505

**Published:** 2025-11-26

**Authors:** Elham Hooshyarazar, Hoda Azizi, Parvaneh Layegh, Maliheh Dadgarmoghaddam, Amir Hooman Kazemi, Seyed Kazem Farahmand, Leili Hafizi

**Affiliations:** ^1^ Acupuncture Department, School of Persian and Complementary Medicine Mashhad University of Medical Sciences Mashhad Iran; ^2^ Radiology Department, Faculty of Medicine Mashhad University of Medical Sciences Mashhad Iran; ^3^ Community Medicine Department, Faculty of Medicine Mashhad University of Medical Sciences Mashhad Iran; ^4^ Department of Traditional Medicine, School of Persian Medicine Tehran University of Medical Sciences Tehran Iran; ^5^ Supporting of the Family and the Youth of Population Research Core, Department of Obstetrics and Gynecology, Faculty of Medicine Mashhad University of Medical Sciences Mashhad Iran

**Keywords:** acupuncture, complementary therapies, integrative medicine, leiomyoma, uterine fibroids

## Abstract

**Background and Aims:**

Uterine fibroids are noncancerous growths of myometrium cells. Seeking for advantageous methods to alleviate their debilitating symptoms is of extreme importance. Acupuncture with global reach could be a beneficial treatment method for uterine fibroids. As in clinical practice, the selection and combination of acupoints is a challenging issue, we conducted a literature review to discover the most frequently used acupoints in treatment of uterine fibroids and the underlying principle of their usage.

**Methods:**

Four databases (PubMed, Cochrane, Scopus, and Google Scholar) were searched for the studies on treating uterine fibroids with acupuncture, restricted to English language and available full texts or abstracts from their inception until January 31, 2025.

**Results:**

Totally, 24 acupuncture prescriptions were found and explored. 53 meridian acupoints, 4 extra points, one ear acupuncture point, and seven Tung's acupuncture points were used. CV4 (Guanyuan), SP6 (Sanyinjiao), Zigong (EX‐CA1) and CV3 (Zhongji) were most frequently selected. The total application of acupoints was 179 times. The acupoints on the Conception Vessel (CV) and Stomach meridian of foot‐Yangming were most frequently applied (30.16% and 16.75% respectively), while the Bladder meridian of foot‐Taiyang had the maximum number of prescribed acupoints (13.84%). The acupoints located on Yin meridians were more frequently used than those on Yang meridians (55.30% vs. 31.84%).

**Conclusion:**

The results of this review can provide a reference of acupoint selection for future surveys. Considering the lack of adequate clinical trials on treatment of uterine fibroids with acupuncture, additional investigations are needed to increase the evidence targeting the efficacy of acupuncture for the management of uterine fibroids.

## 1 Introduction

Uterine fibroids (leiomyomas, myomas) are a prevalent health issue that many women in their childbearing years encounter. These benign masses stem from the smooth muscle of the uterine wall and are influenced by multiple factors [1, 2]. Genetic abnormalities and race play a significant role in the development of uterine fibroids (UFs) [2, 3, 4, 5, 6, 7], with a threefold increase in risk for individuals with a first‐degree family member affected by the condition [8]. Moreover, black women are three times more susceptible to developing UFs than white women [2, 6]. Obesity is a contributing factor to the development of UFs [1, 2, 3, 6], with a higher prevalence observed in individuals who are overweight. Nevertheless, it is essential to recognize that even thin individuals can experience this condition. Based on UFs' hormone‐dependent characteristic [4, 5, 9, 10], their formation initiates with the onset of menarche, and after menopause, there is a possibility of their shrinkage [1, 10, 11, 12, 13, 14]. Furthermore, their incidence is higher among nulliparous individuals [1, 2, 4, 8].

It is estimated that approximately 50% of people with UFs experience symptoms [15]. Different symptoms can arise from UFs, with the specific manifestations dependent on their number and size, as well as their location within the uterus [1, 6, 9, 16]. When a UF is situated underneath the endometrium or in proximity to the uterine cavity, it can lead to abnormal uterine bleeding. UFs located underneath the outer layer of the uterus exert pressure on adjacent pelvic organs leading to pelvic pressure and pain, frequent urination, urinary retention, and constipation [5, 6]. UFs may protrude from the cervix causing postcoital bleeding and dyspareunia. Cervical canal or fallopian tubes obstruction by UFs may lead to infertility [17]. The presence of UFs during pregnancy can cause premature birth, miscarriage, placental abruption, rupture of the membranes, and disruption of the delivery process, whether natural or cesarean [1, 2, 5, 18, 19].

Treatment options for patients are determined by a range of considerations depending on patients' age, their complaint, and the desire to retain fertility [1, 20, 21]. Conventional medicine categorizes UFs treatments into two main groups of pharmacological and surgical interventions [6]. Pharmacological treatments consist of hormonal and nonhormonal drugs [6, 13, 21], typically prescribed for a limited time, provide temporary effectiveness, and often accompanied by various side effects such as medical menopause, bone loss, hepatic toxicity, androgenic effects, and weight gain [9, 13, 16, 22, 23]. Concerning these health challenges tied to drug approaches, the pharmacological interventions must also take into account the role of computer‐aided interventions, which is officially recognized as a noninvasive screening technique advantageous for design, discovery, development, and even repurposing of pharmacological drugs [24, 25, 26]. Surgical approaches are primarily classified into two categories: uterine‐preserving procedures such as myomectomy; and those that involve the removal of the uterus (hysterectomy). While the effect of myomectomy is not permanent and has the possibility of recurrence [6, 16, 22], hysterectomy is associated with the risk of severe bleeding, long‐term hospitalization, postoperative infections, and psychological burden [16, 27].

Considering the complications associated with conventional therapeutic methods, there is a notable shift towards nonpharmacological and nonsurgical interventions to mitigate probable side effects and maintain fertility. Additionally, negative impacts of UFs on females' private and social life [5, 13, 16] necessitate the importance of alleviating symptoms using approved medical approaches.

Acupuncture, with international recognition and endorsement has already treated various diseases and conditions and could be suggested as a beneficial treatment option for UFs. Despite the above‐mentioned fact, there is insufficient published evidence focused on the treatment of UFs with acupuncture. Consequently, gathering and analyzing the accessible and published data from successful available studies seems to be essential to create an available evidence‐based acupuncture protocol for future surveys and integration of acupuncture into conventional medicine for treatment of UFs. As in clinical practice, the selection and combination of acupoints is a challenging issue; we conducted a literature review to discover the most frequently used acupoints in the treatment of UFs and the underlying principles of their usage.

## 2 Methods

### 2.1 Literature Search Strategy

Four major English databases (Pubmed, Cochrane, Scopus, and Google Scholar) were searched for the studies on treating UFs with acupuncture and its variations, restricted to the English language and available full texts or abstracts, from their inception until January 31, 2025. The following keywords were incorporated in this search: (1) “leiomyoma” OR “uterine fibroids” OR “fibroma” OR “myoma” AND (2) “acupuncture” AND (3) “electroacupuncture” AND (4) “acupoint thread embedding”.

### 2.2 Study Selection

#### 2.2.1 Inclusion Criteria

The inclusion criteria for this study encompassed clinical trials, pilot studies, case series, and case reports that investigated the effectiveness of acupuncture and its related techniques (electroacupuncture, scalp acupuncture, auricular acupuncture, fire needling, thread embedding acupuncture and pharmacopuncture) as independent therapeutic method or with additional interventions (e.g., Moxibustion, conventional medications, Chinese herbs, somatic therapies) for treatment of UFs in human adult females diagnosed with UFs, irrespective of randomization or control measures.

Eligibility for trials was based on the reporting of at least one clinical outcome measurement associated with UFs, such as abnormal uterine bleeding, dysmenorrhea, or myoma size, which revealed statistical differences between pre‐ and post‐acupuncture treatment.

Controlled trials were included if their outcomes demonstrated that patients receiving acupuncture experienced significant benefits than those who did not receive acupuncture. The control interventions included conventional medicine and Traditional Chinese Medicine (TCM) herbs.

#### 2.2.2 Exclusion Criteria

Animal experiments, systematic reviews, reviews, and meta‐analyses were not considered for inclusion. However, relevant articles discovered among their reference lists were searched and included if eligible. Trials incorporating acupressure, massage, and transcutaneous electrical nerve stimulation on acupoints as main therapeutic method were also excluded to restrict the emphasis on acupuncture.

### 2.3 Screening and Data Collection

The titles and abstracts of all records obtained from the literature searches were screened independently by authors to discard those that were irrelevant regarding inclusion and exclusion criteria. Authors then compiled, re‐evaluated, and critically analyzed the full texts of the remaining relevant studies. In cases of duplicate publications, the most recent version was selected.

Details of the studies, including titles, journals, authors, participants' information, interventions, controls, main acupoints, outcomes, and adverse effects were extracted and documented in the self‐designed forms. In case the study involved multiple prescriptions, it is essential to extract each prescription separately. The nomenclature of acupoints was standardized in accordance with the Standard Acupuncture Nomenclature [28] by the World Health Organization (WHO) due to the presence of alternative names for acupoints. This study is a narrative review of available published literature, and no human body or animals participated in the study; hence, ethical approval or informed consent is not compulsory.

**Figure 1:**
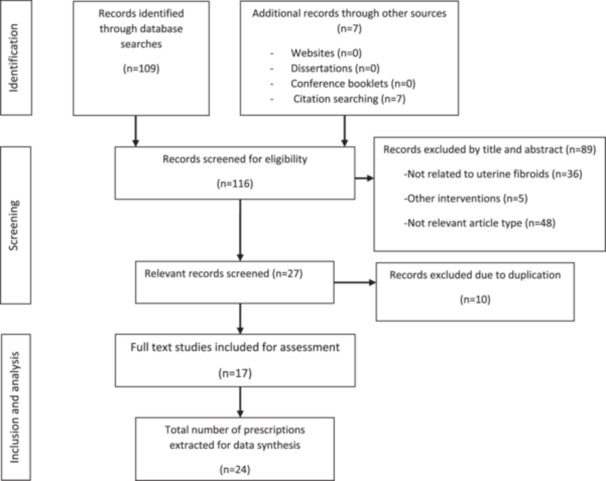
Flowchart of the study selection and data extraction process.

Figure 1. Demonstrates the flowchart of the study selection and data extraction process.

## 3 Results

**Figure 2:**
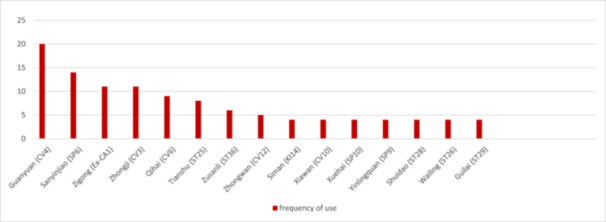
The 16 acupoints are applied with the highest frequency.

**Figure 3:**
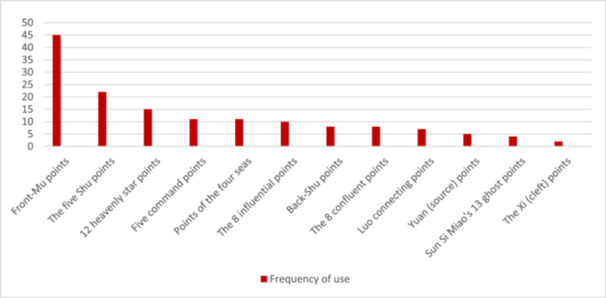
Frequency of use of acupoints in specific categories.

1. Overview of acupuncture prescriptions: A total of 116 records were recognized. Following the screening, evaluation, and elimination of duplicate entries, 17 articles [29, 30, 31, 32, 33, 34, 35, 36, 37, 38, 39, 40, 41, 42, 43, 44, 45] comprising 24 prescriptions were discovered and incorporated into this study. Fifty three meridian‐acupoints distributed over 13 meridians, 4 extraordinary acupoints, 1 auricular acupoint, and 7 Tung's acupoints were recorded for 179 times (157, 14, 1, and 7 times, respectively) in acupuncture treatment for UFs.
2. Acupoints application: The sixteen acupoints applied with the highest frequency are illustrated in Figure 2. Guanyuan (CV4), Sanyinjiao (SP6), Zigong (Ex‐CA1), Zhongji (CV3), and Qihai (CV6) were the top five acupoints in application frequency and were recorded for 20, 14, 11, 11, and 9 times, respectively.
3. Meridians application: The acupoints on the Conception Vessel (CV) and Stomach meridian of foot Yangming were most frequently applied (30.16% and 16.75% respectively), while the Bladder meridian of foot Taiyang had the maximum number of prescribed acupoints (13.84%). The frequencies and proportions of the utilized meridians along with the distribution of the acupoints associated with each meridian are detailed in Table 1. The acupoints located on Yin meridians were more frequently used than those on Yang meridians (55.30% vs. 31.84%).
4. Specific acupoints application: Out of the total 65 utilized acupoints, specific acupoints accounted for 37 (56.92%). The application of specific acupoints was 96 times, which constitutes 53.63% of the cumulative application rate of all acupoints. Indirect calculation strategy was used to determine the utilization rate of specific acupoints by analyzing the utilization frequency of nonspecific acupoints using the formula: ((total application rate − nonspecific acupoints' application rate)/total application rate) × 100%. This method helps to remove any duplication that may result from directly computing the application frequency of specific acupoints, because some specific acupoints fall under two or more classifications due to their unique characteristics.
  Top four most frequently employed specific acupoints' categories were Front‐Mu points, The Five Shu Points, 12 Heavenly Star Points, and The Five Command Points, respectively (Figure 3), while the categories of The Five Shu Points, Back‐Shu points, The 8 Confluent points, and 12 Heavenly Star Points had the maximum number of utilized acupoints. In Table 2, the number of acupoints and application frequencies of specific categories of acupoints are displayed.
5. Acupoints combinations: The assessment of acupoint combinations within prescriptions containing two or more acupoints in their treatment protocols was conducted, and the results regarding these combinations are summarized in Table 3. The most frequently utilized ternary combination of acupoints was assigned to the sets of (SP6, CV4, CV3), (SP6, CV4, Zigong) and (CV4, Zigong, ST25); and the pairwise combination of acupoints that were applied frequently were (CV4, SP6), (CV4, CV3) and (CV4, Zigong) which had the top three supports. Furthermore, the top support for sets of (SP6, CV4, CV3), (SP6, CV4, Zigong), (CV4, SP6), and (Zigong, SP6) emphasizes the significant impact of the local‐distal combination principle in acupuncture treatment protocols.

**Table 1:** Statistics of acupoints on different meridians.

Meridian	Frequency of use	Percent	Number of used acupoints	Percent	Acupoints
CV	54	30.16%	8	12.30%	CV1(1), CV2(3), CV3(11), CV4(20), CV5(1), CV6(9), CV10(4), CV12(5)
ST	30	16.75%	8	12.30%	ST8(1), ST25(8), ST26(4), ST28(4), ST29(4), ST30(2), ST36(6), ST40(1)
SP	28	15.64%	8	12.30%	SP1(1), SP4(2), SP6(14), SP8(1), SP9(4), SP10(4), SP11(1), SP13(1)
BL	14	7.82%	9	13.84%	BL17(2), BL18(1), BL19(1), BL20(3), BL21(1), BL23(1), BL25(1), BL32(3), BL62(1)
KI	9	5.02%	5	7.69%	KI3(1), KI6(1), KI8(1), KI13(2), KI14(4)
LR	5	2.79%	4	6.15%	LR1(1), LR3(2), LR5(1), LR8(1)
GB	5	2.79%	3	4.61%	GB16(1), GB20(1), GB34(3)
LI	3	1.67%	2	3.07%	LI4(2), LI11(1)
GV	3	1.67%	2	3.07%	GV14(1), GV20(2)
PC	2	1.11%	1	1.53%	PC6(2)
LU	1	0.55%	1	1.53%	LU7(1)
SI	1	0.55%	1	1.53%	SI3(1)
TE	1	0.55%	1	1.53%	TE6(1)

Abbreviations: BL, bladder meridian of foot Taiyang; CV, conception vessel; GB, gall bladder meridian of foot Shaoyang; GV, governor vessel; KI, kidney meridian of foot Shaoyin; LR, Liver meridian of foot Jueyin; LI, large intestine meridian of hand Yangming; LU, lung meridian of hand Taiyin; PC, pericardium meridian of hand Jueyin; SI, small intestine meridian of hand Taiyang; SP, spleen meridian of foot Taiyin; ST, stomach meridian of foot Yangming; TE, triple energizer of hand Shaoyang.

**Table 2:** Statistics of acupoints in specific categories.

Specific category of acupoints	Number of used acupoints	Frequency of use	Selected acupoints
The five Shu points	11	22	SP1(1), SP9(4), LR1(1), LR3(2), LR8(1), KI3(1), LI11(1), TE6(1), SI3(1), ST36(6), GB34(3)
Back‐Shu points	6	8	BL20(3), BL18(1), BL23(1), BL25(1), BL21(1), BL19(1)
The 8 confluent points	6	8	SP4(2), PC6(2), SI3(1), BL62(1), LU7(1), KI6(1)
12 heavenly star points	6	15	ST36(6), LI11(1), LI4(2), LR3(2), GB34(3), LU7(1)
Front‐Mu points	5	45	ST25(8), CV5(1), CV4(20), CV12(5), CV3(11)
Luo (connecting) points	5	7	LU7(1), PC6(2), SP4(2), LR5(1), ST40(1)
Points of the four seas	4	11	ST30(2), ST36(6), GV14(1), GV20(2)
Sun Si Miao's 13 ghost points	4	4	SP1(1), BL62(1), CV1(1), LI11(1)
Five command points	4	11	ST36(6), LU7(1), LI4(2), PC6(2)
Yuan (Source) points	3	5	LR3(2), KI3(1), LI4(2)
The 8 influential points	3	10	CV12(5), BL17(2), GB34(3)
The Xi (Cleft) points	2	2	SP8(1), KI8(1)

Abbreviations: BL, bladder meridian of foot Taiyang; CV, conception vessel; GB, gall bladder meridian of foot Shaoyang; GV, governor vessel; KI, kidney meridian of foot Shaoyin; LI, large intestine meridian of hand Yangming; LR, liver meridian of foot Jueyin; LU, lung meridian of hand Taiyin; PC, pericardium meridian of hand Jueyin; SI, intestine meridian of hand Taiyang; SP, spleen meridian of foot Taiyin; ST, stomach meridian of foot Yangming; TE, triple energizer of hand Shaoyang.

**Table 3:** Most frequently used acupoint combinations.

Combination of acupoints	Frequency
Ternary combination of acupoints	
CV4, SP6, CV3	6
CV4, SP6, Zigong	6
CV4, Zigong, ST25	6
CV4, SP6, ST36	5
CV4, CV6, CV12	5
CV4, CV3, CV6	5
Pairwise combination of acupoints	
CV4, SP6	11
CV4, CV3	10
CV4, Zigong	10
CV4, ST25	8
Zigong, SP6	8
CV4, CV6	8
SP6, CV3	7
CV4, ST36	6
CV3, CV6	6
Zigong, ST25	6
SP6, ST36	5
CV4, CV12	5
CV6, CV12	5

Abbreviations: CV, conception vessel; SP, spleen meridian of foot Taiyin; ST, stomach meridian of foot Yangming; Zigong, Ex‐CA1.

## 4 Discussion

UFs lead to considerable physical and psychosocial discomfort for women of reproductive age. Regarding the probable side effects of pharmacological and surgical options to confront these issues on one hand and endeavoring to advance the success rates of treatments on the other hand, complementary medicine, of which, acupuncture is one of the well‐known and commonly practiced forms, has captured the interest of both patients and medical practitioners.

In the attempt to achieve the optimized therapeutic outcome in clinical practice of acupuncture, the appropriate evidence‐based selection and combination of acupoints considering their indication of use, physiological functions, alone or in combination with other acupoints, and patients' body response to stimulation of different acupoints is a challenging issue.

Our results revealed that acupoints distributed on the conception vessel (CV), the stomach meridian of foot Yangming, and the spleen meridian of foot Taiyin were utilized most frequently, while the Bladder meridian of foot Taiyang had the maximum number of prescribed acupoints. Altogether, the acupoints located on Yin meridians were selected in a higher proportion.

Research indicates that multiple factors and theories associated with the development and clinical manifestations of UFs are influenced by acupuncture:

### 4.1 TCM and Acupoints' Theory

TCM theory believes that acupuncture can activate the body's self‐regulatory functions to improve organ disorders, disharmonies, and visceral function in various diseases [8, 11, 29, 46, 47]. According to TCM texts, the main pathologic basis for UF formation is blood stasis [48, 49], and acupuncture can remove this stasis by regulating Qi, promoting blood circulation and dispersing knots [10]. TCM textbooks on gynecology detail treatment strategies for numerous menstrual irregularities, including short, prolonged, or inconsistent cycles as well as insufficient or excessive bleeding [50], which are frequently experienced by individuals with UFs. Moreover, acupuncture and electroacupuncture (EA) have demonstrated superior effectiveness over NSAIDs or no acupuncture controls in alleviating pain associated with dysmenorrhea, a common symptom linked to UFs; however, it was reported to be insignificant when compared to sham or placebo controls [51].

The specificity of acupoints is another important factor in acupuncture trials. There is a prevailing belief that acupuncture at acupoints yields a superior therapeutic effect than non‐acupoints [51]. Research indicates that Guanyuan (CV4), Sanyinjiao (SP6), Zhongji (CV3), and Qugu (CV2) are the most favored acupoints for treating gynecological conditions [34]. Given the tight neurological connection of Guanyuan (CV4) and Sanyinjiao (SP6) to the uterus, their thermal stimulation has been found to improve uterine microcirculation, boost uterine blood flow, and lower vascular resistance (*p* < 0.05) compared with a waiting list group and a healthy group [52]. Zusanli (ST36) stimulation via autologous blood injection nourishes Qi and blood, regulates the hypothalamic‐pituitary‐ovarian (HPO) axis, and promotes the endocrine functions of the pituitary and ovaries compared to the control group (*p* < 0.05) [53], while exhibiting anti‐inflammatory characteristics [54]. Research indicates that mast cell density at Zusanli (ST36) is higher than at sham acupoints in rats. Acupuncture enhances mast cell degranulation, essential for its efficacy, while inhibiting this process may reduce its analgesic effects [55]. It is asserted that electroacupuncture at these acupoints can modulate the reproductive axis's function and facilitate endometrial and uterine morphology repair [56].

According to study findings, manual acupuncture at the Hegu (LI4) and Sanyinjiao (SP6) acupoints bilaterally, with involvement of the autonomic nervous system, may contribute to the management of dysmenorrhea [50, 57], which is a key marker of irregular menstrual flow, a typical prevalent manifestation of UFs.

### 4.2 Neurochemical Effects of Acupuncture

The nervous system is a crucial determinant of the intricacies of human life. The principles of neurophysiology provide additional insights into acupuncture mechanisms. Needle insertion would activate local receptors, transmitting signals to the spinal cord and brain to regulate physiological tasks [51]. A systematic review and a narrative review investigating brain responses to acupuncture using functional magnetic resonance imaging show stimulation of major brain integration centers by acupuncture [58, 59, 60]. In the proceedings of two acupuncture congresses, as well as in a clinical trial, it was asserted that acupuncture can adjust nerve functions and improve the performance of internal organs and hormone secretions [46]. Female hormones are regulated by acupuncture through neuroendocrine pathways via engaging autonomic afferents to signal the central nervous system; however, clinical trials with larger sample size and a sham acupuncture control group without needling is needed to distinguish this effect from parasympathetic enhancement due to relaxation strategies employed in acupuncture facilities before and during intervention [29].

Based on various reviews and a randomized controlled trial protocol, acupuncture has the potential to enhance the expression of neuropeptide genes. EA with different frequencies, such as 2 Hz and 100 Hz produces diverse impacts on opioid gene expression with distinct neuronal pathways. Acupuncture and electroacupuncture activate sympathetic nerve fibers to enhance migration of opioid‐containing cells to the site and stimulate neurochemical release, notably endogenous opioids (beta endorphins, enkephalins, and dynorphins) together with serotonin and dopamine neurotransmitters [46, 47, 61, 62, 63]. The release of opioids and neurotransmitters provides pain relief, promotes sedation, and support the recovery of motor functions, relevant for menstrual pain management [50, 64], which is the most commonly documented symptom of UFs [65]. It is assumed that when acupuncture needles are inserted or electrical stimulation is applied, adenosine triphosphate (ATP) is released from skin keratinocytes and fibroblasts, which rapidly breaks down into adenosine, binds to adenosine A1 receptors, leading to the analgesic effect of acupuncture [66]. Furthermore, visceral and somatic signals triggered by acupuncture towards the central nervous system promote anti‐inflammatory responses through humoral and neural pathways [50, 64]. Yet, the overall low quality of methodology makes it difficult to draw a definitive conclusion, which highlights the need for well‐designed, high‐quality, randomized, double‐blind trials with various types of control groups.

Results of a feasibility study supported the central effect of acupuncture on UFs and related symptoms because the study didn't utilize local acupoints for treatment of UFs (*p* < 0.05 for 10 out of a total of 14 items) [29]. Concerning the study's limited sample size and absence of a control group and blinding, this claim may be uncertain, and additional research is required to verify its legitimacy, necessitating the execution of larger clinical trials incorporating randomized negative controls.

### 4.3 Hormonal Effects of Acupuncture

Uterine fibroid development is influenced by complex feedback mechanisms between sex hormones (estradiol and progesterone) and growth factors, with a higher incidence in women with elevated levels of these hormones [11, 29, 67]. Existing therapies focus on modulating estradiol and progesterone synthesis or action [9]. Literature reviews indicate that acupuncture can regulate these hormones [62]. Studies show that acupuncture may play a substantial role in reducing elevated serum estradiol levels [68].

Reviews on possible ways acupuncture may influence the reproductive system revealed that acupuncture harmonizes the body's internal regulatory mechanisms and signal transmission in the spinal cord (segmental level), including the sympathetic nervous system, and in the central nervous system (CNS). While the CNS plays a role in controlling hormone secretion from the pituitary gland, acupuncture may regulate the endocrine system and the neuroendocrine system as well [69, 70]. Acupuncture's ability to modulate the neuroendocrine system has gained significant interest in its application in the reproductive field [62].

The regulation of female reproductive functions is significantly influenced by the HPO axis which has also been correlated with the formation of UFs [71]. In light of the theory that acupuncture can regulate the pituitary gland, the thyroid gland, and the central nervous system, without pharmacological interference, it is reasonable to regard acupuncture as a viable therapeutic option for UFs; however, taking into account that no randomized double‐blind controlled trials fulfilled the inclusion criteria of this systematic review, the effectiveness of acupuncture in managing uterine fibroids was reported as uncertain, highlighting the necessity for well‐structured RCTs with long‐term follow‐up [11]. Acupuncture could impact UFs by rectifying the function of the HPO axis, but according to a 2023 systematic review, acupuncture failed to effectively decrease estrogen levels, suggesting that the molecular pathways engaged by acupuncture differ from those targeted by Western medicine, underscoring the need for additional research [71]. Investigations have revealed that acupuncture can modify plasma β‐endorphin levels, subsequently affecting hypothalamic activity and influencing the secretion of Gonadotropin‐releasing hormone (GnRH), Gonadotropin, and other hormones associated with reproductive function [50, 62, 72, 73] by applying a tonic inhibitory effect on the GnRH pulse generator [69] ultimately regulating the HPO axis and the menstrual cycle. An experimental animal study showed that repeated low‐frequency EA can led to a rise in hypothalamic β‐endorphin in rats [74].

Application of acupuncture on connective tissue can convey mechanical signals to the regulation of connective tissue metabolism, potentially serving as an essential element in the acupuncture treatment of uterine fibroids [46].

### 4.4 Acupuncture and Uterine Blood Flow

Clinical trials and animal studies indicate that acupuncture influences uterine and ovarian blood flow through a reflex response involving ovarian sympathetic nerves, controlled by supraspinal pathways [75, 76, 77]. EA exhibits a selective targeted effect on blood flow increase or decrease in specific organs if a proper combination of dermatome and stimulation frequency is employed. Evidence suggests that only high‐frequency EA is capable of decreasing ovarian blood flow in rats. Moreover, the neuroanatomical pathways responsible for this process encompass both central mechanisms and segmental innervation [33, 78, 79, 80].

Based on investigations of a case study and a case series, high‐frequency (80‐Hz) EA at Sanyinjiao (SP6) and Guilai (ST29) in the follicular phase of the menstrual period led to decreased blood flow in the uterine arteries bilaterally (*p* < 0.05) [33, 81], subsequently alleviating menorrhagia in a patient suffering from UF [33]. However, these studies did not explore the consequences of long‐term high‐frequency EA and the enduring aftereffects of it on the blood flow of uterine arteries, emphasizing the need for larger‐group, randomized controlled investigations in both follicular and luteal phases regarding their specific blood flow dynamics. A randomized controlled trial featuring the characteristics outlined above is currently being conducted [82].

### 4.5 Acupuncture and Oxidative Stress

Studies point to the involvement of oxidative stress, the imbalance between pro‐oxidants such as reactive oxygen species (ROS) or free radicals, and antioxidants in the development of UFs by affecting genetics, epigenetics, and fibrogenesis through peroxidative DNA damage, the induction of apoptosis, and lipid peroxidation [83]. Recent findings reveal that acupuncture, by boosting endogenous antioxidant enzymes' activity and suppressing pro‐oxidant enzymes, combats oxidative stress damage in various organs and strengthens the immune system [84, 85, 86, 87, 88], hence, by providing an antioxidant effect through free radicals' inactivation, it maintains the immune balance in the endometrial microenvironment [62].

### 4.6 Anti‐Inflammatory Effects of Acupuncture

Reviews confirm that acupuncture can exert anti‐inflammatory effects by affecting the Hypothalamic‐Pituitary‐Adrenal (HPA) axis to lower Cyclooxygenase‐2 (COX‐2) and Prostaglandin E2 (PGE2) [51, 63]. As COX2 inhibitors are indicated for the treatment of dysmenorrhea and menorrhagia [89], this function of acupuncture may be effective for fibroid‐related dysmenorrhea and menorrhagia treatment.

Considering the fact that COX2 can initiate tumor formation and promotion [89] and since the expression of COX‐2 in UFs is higher and the inhibition of COX‐2 activity significantly reduced the proliferation of smooth muscle cells of the UFs [90], it may be legitimate to consider COX2 inhibitory effect of acupuncture as a potential therapy for UFs.

### 4.7 Acupuncture and Growth Factors

Evidence shows that transforming growth factor β (TGF‐β) overexpression is linked to the proliferation of myometrial tissue. Additionally, treatments decreasing TGF‐β signaling have proven effective in diminishing fibroid growth and symptoms as indicated by literature reviews [4], and according to several experimental animal studies, acupuncture can reduce TGF‐β expression in various tissues, especially the uterus [56, 91, 92, 93, 94].

### 4.8 Acupuncture, Obesity, and Uterine Fibroids

Research has indicated increased serum levels of leptin and ghrelin in women with UFs [4]. Acupuncture, as discussed in published reviews, may affect energy homeostasis by influencing neurohormones, particularly leptin, leading to decreased appetite and altered satiety at the hypothalamic level, establishing acupuncture as an effective treatment for obesity, which is associated with an increased risk of UFs [95, 96].

Furthermore, the regulation of Leptin, thyroid gland, and the hypothalamus‐pituitary‐adrenal cortex axis activity through the stimulation of some specific acupoints used for weight loss, can contribute to the normalization of menstrual cycles in obese women [50]. A systematic review of clinical trials has also confirmed the efficacy of acupuncture in regulating menstrual cycles, together with decreasing body mass index in polycystic ovary syndrome (PCOS) [97]. The aforementioned advantages of acupuncture, along with the high occurrence of abnormal and irregular menstrual cycles linked to UFs, indicate that acupuncture may serve as an effective therapeutic option for managing UFs and associated symptoms.

### 4.9 Emotional Effects of Acupuncture

Studies have reported an association between high stress levels and UFs pathogenesis. The hypothesis behind this relationship includes: (1) HPA axis disturbance leading to cortisol and epinephrine release, (2) fluctuations in estrogen and progesterone levels provoked by stress, and (3) systemic inflammation triggered by stress; all three of which are known to be linked with increased UFs risk [2]. Meanwhile, despite the physical impacts of UFs on women's health, these tumors, if symptomatic, can give rise to emotional and psychosocial sequels leading to a diminished quality of life [98, 99].

Results derived from different reviews have demonstrated that acupuncture, by influencing both specific and nonspecific neurological signaling pathways, alongside the regulation of neuromodulators including cortisol, prolactin, epinephrine, beta endorphin, enkephalins, and stress‐related hormones (e.g., adrenocorticotropic hormone), can mitigate depression, anxiety, and stress [50, 61, 95, 100]. Thus, it can be a beneficial option to minimize stress‐related risk of UFs formation and help women with UFs gain control of their lives in managing their fibroid‐related symptoms.

The accelerated progress of information technology has resulted in massive data for acupuncture studies. Despite its notable advantages in clinical practice, the principles underlying these data and scientific standardization, especially concerning the quantification of organ‐level interactions and the measurable impact of acupoint stimulation, have yet to be comprehensively investigated. Exploring computational acupuncture for UF management will reveal an innovative attitude in the acupuncture application aimed at modeling the therapeutic dynamics that can aid in the acupoint selection for UF patients and facilitate clinical decision‐making by proposing personalized acupuncture plans targeting the specific conditions of each patient. Techniques involving three‐dimensional visualization of meridians and acupoints, imaging of nerve and vascular structures that encircle acupoints at high resolution, thermal distribution patterns' study and analysis of bioelectrical signal transmission near acupoints enable the quantitative evaluation of needling effects providing novel perspectives on the physiological mechanisms underlying acupuncture, the estimation of intervention efficacy, determining the optimal combinations of acupoints suited to particular clinical scenarios and increasing the capacity for innovative clinical advancements offering valuable guidance for researchers and clinical professionals [101, 102].

## 5 Conclusion

Our literature review aimed to discover the most frequently used acupoints for treatment of UFs with acupuncture and the underlying principles of their usage to create an efficacious evidence‐based protocol for future trials and integrate acupuncture into conventional medicine for management of UFs. Guanyuan (CV4), Sanyinjiao (SP6), Zigong (Ex‐CA1), Zhongji (CV3), and Qihai (CV6) appeared to be selected more frequently in existing documents and are recommended by authors to be incorporated into fibroid treatment protocols. Acupuncture was noted to target various factors associated with UFs development and symptoms, including neurochemical, hormonal, and inflammation pathways, uterine blood flow, growth factors, and oxidative stress. Regarding the lack of adequate studies in this field, more well‐designed, high‐quality, blinded, randomized clinical trials with larger population sizes and extended follow‐up periods are needed to explore acupuncture impact on multiple risk factors associated with UFs formation and development, as discussed in the text, to provide more standardized and comprehensive acupuncture guides for UFs treatment.

## Author Contributions

**Elham Hooshyarazar:** conceptualization, investigation, writing – original draft, methodology, validation, visualization, writing – review and editing, software, formal analysis, project administration, data curation, resources. **Hoda Azizi:** conceptualization, investigation, validation, visualization, writing – review and editing, data curation, supervision, resources, methodology. **Parvaneh Layegh:** validation, visualization, data curation, supervision, writing – review and editing. **Maliheh Dadgarmoghaddam:** validation, visualization, data curation, supervision, writing – review and editing. **Amir Hooman Kazemi:** validation, visualization, data curation, supervision, writing – review and editing. **Seyed Kazem Farahmand:** validation, visualization, data curation, supervision, writing – review and editing. **Leili Hafizi:** validation, visualization, writing – review and editing, project administration, data curation, supervision.

## Conflicts of Interest

The authors declare no conflicts of interest.

## Transparency Statement

The corresponding author, Leili Hafizi, affirms that this manuscript is an honest, accurate, and transparent account of the study being reported; that no important aspects of the study have been omitted; and that any discrepancies from the study as planned (and, if relevant, registered) have been explained.

## Data Availability

The data are available on request from the corresponding author.
